# Supplementary material for: Thunderstruck: The ACDC model of flexible sequences and rhythms in recurrent neural circuits
Source: PLoS Comput Biol. 2022 Feb 2;18(2):e1009854. doi: 10.1371/journal.pcbi.1009854 (PMC8843237; doi:10.1371/journal.pcbi.1009854)
Supplement: Attachment — Submitted filename: Cover letter.pdf [file pcbi.1009854.s005.pdf]

Dear Dr. Gershman,

We thank you, Dr. Logiaco and the anonymous Reviewer for the positive appraisal of our paper and the useful comments that have allowed us to clarify both the contribution of our paper with respect to previous work and the inner workings of our model. Below you may find a point-by-point reply (in green font color) to the comments of both Reviewers. Where applicable we have made changes to our original manuscript, and, as requested, have resubmitted both a version with the changes highlighted (in green) and a clean version of our revised manuscript. We believe the comments of the Reviewers have undoubtedly increased the quality of our manuscript, and we hope the current version is adequate for publication in *PLOS Computational Biology*.

Cristian B. Calderon
Tom Verguts and
Michael J. Frank

Reviewer #1:

Major Points

** This article describes a model where a cortical module, a basal ganglia module, and a thalamic module interact to perform action selection at adjustable timings. Specifically, the model combines a chain-like structure that is distributed over the three modules and that encodes the sequence order, while the modulation of action timing is implemented through dedicated reciprocal thalamo-basal connections. The larger structure of the network is hard-wired, while some adjustable synaptic weights are learned through local learning rules and simple scalar reinforcement signals.*

** The biggest strength of the paper is its very engaging narrative, and the large effort put into showcasing the functionality of the network with nice detailed figures, videos, and code.*

We thank Dr. Logiaco for her positive appraisal of our paper and her recognition of the effort we put to showcase the model's functionality through dynamical animations. Beyond its engaging narrative etc, we believe our paper does present a model that possesses unique functionalities (both computational and links to empirical data), as elaborated below.

** I am not confident about the actual extent of the relative computational advantages of the proposed model - but I don't think that this should preclude publication in Plos CB as this model does have some specific design elements that do work.*

-First, state-space models are actually able to do temporal rescaling (Flexible timing by temporal scaling of cortical responses, Wang et al. Nat. Neuro 2018) and can be trained to behave well in a variety of settings using continuous reinforcement signals (Recurrent neural networks as

versatile tools of neuroscience research, Barak, Curr Opin Neurobiol, 2017), including by local learning rules (Predicting non-linear dynamics by stable local learning in a recurrent spiking neural network, Gilra and Gerstner Elife 2017; Local online learning in recurrent networks with random feedback, Murray, Elife 2019) that can sculpt a diversity of connectivity structures (Emergence of functional and structural properties of the head direction system by optimization of recurrent neural networks, Cueva et al, ICLR 2020). Therefore, the relative advantage of the currently suggested model lies in its ability to flexibly perform temporal operations while only being trained with simpler learning rules.

We agree that state-space models (e.g., Wang et al. 2018, Nat. Neuro; Egger & Jazayeri, 2020, Nat. Commun.) can perform temporal rescaling by changing the amplitude of (one of) the inputs to the RNN. However, these models either addressed scaling of only a single action (Wang et al. 2018, Nat. Neuro), single action sequences without investigating potential learning mechanisms (Egger & Jazayeri 2020, Nat. Commun.), or with a very specific learning mechanism of which it is unclear if it can learn and rescale both synchronous ***and*** asynchronous action sequences[1]. While various extensions of these models could be investigated, the learning and stability of such mechanisms is yet to be uncovered. Our model extends these models by naturally scaling both types of sequences (synchronous ***and*** asynchronous) without retraining. We have now clarified this in the introduction, and have acknowledged the ability of state-space models to perform some level of temporal rescaling.

Furthermore, we acknowledge the important effort made in the field to provide biologically plausible and local learning rules for RNNs. In fact, prior to developing this work, we have dissected and implemented one of these rules presented in the work of Miconi (2017, eLife). Whereas these learning rules possess the advantage of being local, they come at a cost in terms of the complexity of the target function that can be learned by the model. Furthermore, it is unclear whether these learning rules can account for flexible temporal rescaling of either synchronous or asynchronous sequences.

We have revised our manuscript in light of this comment, and write on line 118:

---

[1] First, in the Wang et al. model, temporal rescaling only involves a single action, i.e. speeding up or slowing down the interval between a go cue and a single appropriate motor output time. It is unclear how this would apply to temporal rescaling for a sequence of actions. One could imagine extending the model whereby the output of one RNN serves as the input to a successive RNN, but both learning and stability of such a potential network have yet to be investigated. Second, Egger & Jazayeri (2020, Nat. Commun.) propose a state-space model involving two inhibitory neurons projecting to a leaky integrator. This model is able to perform temporal rescaling on single action sequences by changing the amplitude of the input (lower inputs lead to faster sequences). Still, although flexibility involves quick adaptation of the dynamical system (via changing the input amplitude), the sequence has to be initially learned and this model does not propose a learning mechanism for the sequence. Hardy et al. (2018, Nat. Commun.) proposed a high dimensional RNN (i.e. more than two inhibitory neurons) that learned to display temporal rescaling (again on a single action sequence). Crucially however, their model needed a very specific learning regime to display this property. Moreover, none of these models can perform temporal rescaling on both synchronous ***and*** asynchronous (where the inter-action-interval is not constant) sequences involving distinct actions.

"[…] Recent work has shown that biological learning rules using local information can effectively learn complex (sequential) tasks[42–44] (albeit not as effectively as non-biological rules). State-space models can also implement a rudimentary form of temporal rescaling, in that they can rescale the timing of the execution of a single motor response[45], and iso-synchronous action sequences (e.g., index tapping at a steady rhythm)[34]. However, these models do not support temporal rescaling in the more general case (i.e., asynchronous action sequences). Furthermore, given their focus on cortical networks, these models do not address the growing evidence that action sequences unfold over multiple levels within cortico-basal ganglia-thalamic loops, with attractor state switches occurring in the prefrontal cortex[46] and action timing represented in the basal ganglia[2,4]"

-Second, it looks like ring-like models (e.g. Recurrent networks with short term synaptic depression, York and Van Rossum, J Comput Neurosci 2009; Learning multiple variable-speed sequences in striatum via cortical tutoring, Murray and Escola, Elife 2017) could perform very similar operations as the proposed network. Indeed, such ring-like models can be delayed by not providing excitation to the circuit (resulting in a silent network). In addition, in such models, larger background currents lead to faster dynamics, so these models support temporal rescaling. Finally, 'temporal compositionality' (i.e. following a higher-level rhythm signals) could be achieved by using a 'rhythm command' consisting of a time-varying background input (using larger inputs when wanting a fast interval, and smaller inputs when wanting to slow down the rhythm). The relative advantage of the currently proposed model is therefore to suggest how to achieve these types of operations using a larger cortical-basal-ganglia-thalamic network, and using a 'rhythm command' that consists of timed pulses (as shown in fig. 7b) instead of the above mentioned time-varying background input.

We thank Dr. Logiaco for this very relevant comment. We do agree that ring-like models are able to perform temporal rescaling, often implemented as changes in the background current, which could also be controlled by the rate of synaptic depression (higher rates would lead to faster dynamics). However, the later would require different time constants of synaptic depression for different rates of rescaling (i.e. not a very biologically plausible mechanism). We also agree with the fact that not providing excitation for a certain amount of time would result in a silent network that can then be reactivated when the background current is reinitiated. However, this proposal would need an extra assumption in order to produce robust sequences. Indeed, following this proposal it is not straightforward for the network to ensure that the next action in line is produced. If the network is fully silent, inherent noise in the network will randomly select which unit (or cluster of units) is activated next in line, given that background current is global. Therefore, this solution would require additional higher-order knowledge of where the sequence was stopped to ensure that the sequence order is respected. While this could perhaps be controlled by self-recurrent connections in the RNN, this would interact with synaptic depression and may not be robust, especially to different spacing between actions.

Re: temporal compositionality, we also agree that a 'rhythm command' that dynamically controls the strength of the background current could be useful. However, ring-like models are only able to learn synchronous sequences (i.e., with fixed inter-action intervals). While a

rhythm command could be used to produce asynchronous sequences, it is unclear how the ring model could learn asynchronous sequences ***and*** apply temporal rescaling on these sequences. Our hypothesis, instantiated in our model, is that the brain leverages distinct mechanisms to be able to learn any arbitary asynchronous sequence and apply any rhythm (including rescaling) to those sequences -- that is, to achieve full flexibility.

Our revised version discusses these issues in the discussion section of our paper (line 570):

"Nevertheless, ring-like models with synaptic depression[25,90] could potentially account for these properties. Indeed, temporal rescaling in these models is often implemented as changes in the background current (higher current levels lead to faster rescaling). Therefore, to produce asynchronous sequences, one could imagine a dynamical background current which is null when no action has to be provided, resulting in a silent network, and turned on when the sequence has to be resumed. However, given a silent noisy network, reactivation of a global current will induce activation of a random cluster in the sequence and not necessarily the next cluster in line[2] (this is footnote #3 in the main text), thereby not displaying robustness in sequence production.

Line 580: […]. Our model proposes a more robust approach to sequence flexibility. The sequence in our model is chained by the execution of the previous action, thus our model does not require an explicit representation of the most recent action (see footnote 3). Indeed, what controls the sequence is a series of local signals (i.e. selective feedback from the motor thalamus to the RNN[49]) rather than a global signal, and what controls the timing is a global input to the BG. This feature is key for our model to account for the distinct types of flexibility described in the results."

** Some comparisons are made with electrophysiology and behavior, and the authors demonstrate that some basic features of neuronal activity are consistent with the model.*

*-One point that I think should be clarified is that the model focuses on action *selection* rather than action execution. Indeed, even for simple actions such as 'playing the piano' as suggested by the authors, the muscle commands that need to be generated by the brain are continuous (A neural network that finds a naturalistic solution for the production of muscle activity, Sussillo et al, Nature Neuroscience 2015; Motor Cortex Embeds Muscle-like Commands in an Untangled*

---

[2] Indeed, for such a system to be viable, the network would need an additional piece of information, which is a memory of which cluster (or unit) was last active. The reason is that, in ring-like models the background current is global (i.e., sent to all units). Thus, if the network goes silent, reactivation of the network would not ensure that the appropriate cluster (i.e. the one next in line) becomes active; i.e. noise would randomly activate a cluster and the sequence would restart from that cluster (see implementation of [25]). Consequently, some extra input signal (representing a memory of which cluster was last active) should give an advantage to the next cluster in line, in order to ensure that sequence order is maintained. This extra input signal could take the form of recurrent connections within the cluster, controlling the activation decay of that cluster such that it would still be active after long periods of time (as opposed to all other clusters that would be fully silent; see [19]). Note however that recurrent connections would put a break on the synaptic depression, as these two parameters trade off; therefore, formal implementation of this proposal would be needed to understand the model regimes.

Population Response, Russo et al., Neuron 2018; Thalamic control of cortical dynamics in a model of flexible motor sequencing, Logiaco et al., Cell Reports 2021).

We agree with this point; our model does indeed focus on action selection rather than execution. We now have clarified this point in the discussion; we write on line 586:

"It is important to clarify that flexibility in our model is implemented at the action selection level rather than action execution or implementation. Indeed, although we use musical examples to motivate our work, our model focuses on the timing/selection of actions rather than their execution. Even simple finger movements implemented to play the piano require muscle commands represented as highly dimensional and continuous signals[40,107,108]".

*-It would be nice to mention that the involvement of basal ganglia in *fixed order* sequences of movement is a debated topic (Motor learning, Krakauer et al., Compr. Physiol. 2019, available here: https://www.researchgate.net/profile/John-Krakauer/publication/331774116_Motor_Learning/links/5c90db8b45851564fae71871/Motor-Learning.pdf).*

We thank Dr. Logiaco for this relevant comment and point on line 65 that the role of the basal ganglia in sequence learning is a debated topic, referencing the work of John Krakauer. However, we note that there are many pieces of data suggesting BG coding of action sequences (e.g. Jin & Costa 2015 for review). Moreover, disrupting striatal activity causally alters action selection (e.g., Doi et al 2020). Furthermore, sequence learning requires striatal NMDA receptors, and during sequence production, optogenetic stimulation of the direct and indirect pathways results in inserting an extra action element or deleting an action segment within a sequence, respectively (Geddes et al., 2018).

-It could be nice to mention that some evidence suggests that neurons at the output nuclei of basal ganglia have a more categorical type of response rather than an accumulation type of response as suggested by the model (Basal ganglia subcircuits distinctively encode the parsing and concatenation of action sequences, Jin et al., Nature 2014).

See reply to the next comment.

-It would be nice to discuss that the direct connections from basal ganglia to thalamus are thought to be mostly inhibitory - but disinhibition is possible (through the thalamic reticular nucleus or more directly, e.g. Inhibitory Basal Ganglia Inputs Induce Excitatory Motor Signals in the Thalamus, Kim et al., Neuron 2017).

We thank Dr. Logiaco for these interesting comments and we reply to both of them here.
The BG component of the model described here summarizes the contributions of more detailed models of BG circuitry in our previous models applied to reinforcement learning and decision making settings (e.g, Frank, 2005, 2006; Franklin & Frank 2015). In these models, striatal neurons exhibit accumulation of evidence, but they project through direct and indirect

pathways to influence categorical discrete signals in BG output nuclei, leading to disinhibition of thalamus (see Ratcliff & Frank, 2012; Wiecki & Frank 2013 for examples). These patterns are also observed empirically in terms of striatum accumulation signals and discrete downstream responses in BG output nuclei once a threshold of accumulation is reached (Doi et al., 2020, eLife; Schmidt et al 2012). In the present model, we assume our G units as striatal, evidence accumulation, units, but we abstracted over the rest of the BG circuitry, omitting the disinhibitory signals to thalamus and summarizing their net disinhibitory effect as excitatory (see also O'Reilly & Frank 2006 for a similar abstraction). Furthermore, we discuss how activation of the output nuclei of the BG leads to stronger signals in the motor thalamus; we now write on line 702:

"The BG component summarizes the contributions of more detailed BG circuitry[140,167,172]. In these models striatal neurons accumulate evidence, which via the direct and indirect pathways leads to categorically discrete signals in BG output nuclei, and to disinhibition of the thalamus (e.g., [132,168]). These patterns are also observed empirically in terms of striatum accumulation signals and discrete downstream responses in BG output nuclei once a threshold of accumulation is reached[169]. Here, we lumped together the double inhibition from striatum to Globus Pallidus (GP) and from GP to the thalamus into a single excitatory projection to keep the model simple and tractable. Interestingly, optogenetic stimulation of the GP has been shown to increase the firing rate of motor thalamus neurons[173]."

Minor Points

** The temporal rescaling operation also induces a shift.*

This is true, temporal rescaling induces a very small shift in the sequence. We now acknowledge this property. We view this small shift as an emergent property of a global timing signal in the BG. However, it could be avoided by simply targeting all but the first G node in the network; we now write on line 305:

"Note that rescaling also induces a tiny shift in the sequence. This is an emergent property of a global rescaling signal to all Go nodes of the network; this slight shift could be avoided by targeting all but the first Go node with the multiplicative term."

** Just by reading the main text, I was not sure whether the plastic connections in all parts of the network (recurrent RNN connections, RNN -> go connections; go -> 'action' connections) were learned simultaneously. I think this should be clarified. Also, in Fig. 1, it would be nice to highlight (with a specific color) the plastic connections, and to put symbols to clearly identify the corresponding types of learning rule used.*

The Hebbian learning rule (i.e. equation 1) unfolds on a fast time scale, i.e. within an action sequence simulation. The delta rule unfolds on a slower time scale, i.e. after a sequence is executed sequences (when the supervised signal to learn action timing is received).

Furthermore, as mentioned in the paper, learning is sequential (i.e. one action after the other; see methods). We have now clarified this information in the paper on line 172. Regarding figure 1, we actually had added this information in the simplified architecture figure (left panel of figure 1) in order not to overcrowd the detailed architecture figure. In the simplified architecture, dashed lines represent plastic connections, and the associated rule is written next to the dashed line (i.e. “Hebb” or “Delta”, respectively for hebbian learning and the delta rule). We did however forget to relay this information in the caption of the figure, and have remedied this oversight (see line 206).

** The discussion about the behavioral evidence of learning sequences one item at a time, now present in the Methods, would in my opinion be very useful to include in the main text.*

We have now moved this information in the main text (line 179).

** In Fig. 2, it would be nice to*
-mention explicitly the meaning of the indices (ith cluster, jth ...). Could it be possible to use indices that correspond to 'action selection order'?

We believe this comment refers to the right panel of figure 1 rather than figure 2. The meanings of the indices are described in the methods, but we now have also added them in the captions of figure 1 for clarification.

-in panel A, it would be nice to always use different shades of color to indicate the progression over learning

In fact, this is exactly what is depicted in panel A. The result is not as expected given that most of the curves sit directly on top of the desired action time. Therefore, the change in shade happens in the same location. Indeed, we prefer to show how the model unfolds over the entire span of learning, that is until the last action is learned.

* lines 735-736: for J^{AG}, are you describing the initial weights (before learning)?

Yes, we are. We now clarified this point at the same location.

Reviewer #2:

Major Points

This work proposes a multi-regional model, the associative cluster-dependent chain (ACDC), that learns to flexibly execute actions. The flexibility consists in being able to temporally shift multiple actions in time, recombine them, and scale their duration, even though the network was not explicitly trained on all the possible actions in a supervised manner. The authors also show that with their model they are able to account for behavioral and neurophysiological observations. They then make use of their model’s flexibility to replay a learned song in a

different rhythm. The novelty in this work consists in proposing a biologically plausible model with a wide-ranging amount of flexibility that was not previously seen in a single model. It is also interesting in that it generates some insight into possible computational mechanisms that could give rise to a couple of behavioral and neurophysiological empirical observations.

We thank the Reviewer for her/his positive appraisal of our manuscript and for the relevant comments that allowed us to clarify exactly in what aspects our model is novel and what is its main goal.

However, to fully appreciate the contributions of this work and understand how exactly it compares to and extends previous work, the authors need to enhance clarity in the following general ways. Currently, it is unclear (1) what exactly is the novelty in this work, (2) how the model is trained and works, and (3) to what extent the authors claims are supported by the actual findings (Figures).

The Reviewer's comments are very much in line with those of Dr. Logiaco (Reviewer 1). We have now substantially clarified the novelty in our work, how the model is trained and how it works. Furthermore, we clarify how our claims are supported by the findings.

It is unclear what exactly the novelty in this work is... If it is the biological plausibility, then the authors should carefully spell out what aspects of the model are in fact biologically plausible (e.g. in Introduction). While the overall modular architecture seems to be biologically plausible, some of the training mechanisms are not necessarily so (e.g. supervised training in certain parts of the network; pre-established action modules).

The novelty in our work is twofold. First, at the function level, our model can support several temporal flexibility properties simultaneously (including asynchronous sequences, shifting, rescaling, and compositionality). Existing models support only more rigid versions of a subset of these features. As articulated in the revised manuscript (see line 119), state space models can support rescaling but only for single actions or iso-synchronous sequences. None support temporal compositionality as currently implemented. Second, our model develops an account for the role of corticobasal ganglia loops in flexible sequence learning and production, constrained by empirical neurophysiological data, using simple biologically plausible learning rules. Indeed, the temporal flexibility functions of our model arise from the separation of mechanisms within the circuit, with cortical RNN representing latent states within a sequence, and BG controlling both the timing of the transitions from one state to the next and which actions are linked to sequence positions.

Re: learning rules, in fact, the supervised delta rule is only used at the output of our network to optimize readout weights. We do not view this to be implausible given that we model sequence learning situations in which signed error feedback is provided (as in Kornysheva et al. 2019, Neuron), such as when a tutor teaches you how to play the drums and holds the tempo, or in bird-song learning (where a tutor is available to provide signed error). There are several biologically plausible implementations of the delta rule when such error signals are available

(see e.g., Lillicrap et al., 2016, Nat. Commun.; Lillicrap et al., 2020, Nat. Neuro. Rev.; O'Reilly et al., 2012, Computational Cognitive Neuroscience Book, XCAL model; see line 622 in the discussion).

Re: pre-established action modules, we inherit this assumption from many models of cortico-BG circuits (e.g., Gurney et al, 2001; Frank, 2005, etc) in which cortical motor units are linked to specific actions (e.g. via connectivity to the corticospinal tract), together with anatomical and physiological data showing that this topography is maintained throughout the circuit from cortex to striatum to BG output, thalamus and back to motor cortex (e.g., see Hintiryan et al., 2016; Hunnicutt et al., 2016; Peters et al, 2021; see line 739 in the methods).

We have now clarified the novelty of our work in light of this comment; at the end of the introduction (on line 142) we write:

"Here, we develop a biologically inspired[1] RNN called the associative cluster-dependent chain (ACDC) model. By combining strengths of the associative chain and cluster-based models, ACDC accounts for biological data. As we show below, the novelty in our model is twofold. First, we propose a biologically-plausible model of cortico-basal ganglia-thalamic loops that decomposes the functions of cortex and basal ganglia and learns sequences based on simple local and (biologically motivated) supervised learning rules. Second, this decomposition affords greater flexibility in generating desired action sequences, supporting temporal asynchrony, shifting, rescaling, and compositionality in a single model. Crucially, our model factorizes action sequence features within the circuit, with cortical RNN representing latent states within a sequence, and BG controlling both the timing of the transitions from one state to the next and which actions are linked to sequence positions. Factorizing order and timing information by storing them separately in a premotor cortical RNN, which is dynamically gated by a basal ganglia-thalamus module, affords independent (and flexible) manipulation of sequence order and action timing, and thus increases computational flexibility."

With regards to other work, the authors argue that state-space models, for instance, draw on non-biological learning mechanisms and cannot encode multiple sequences nor exhibit temporal scaling (p.5). But more biologically realistic learning rules have been proposed that are able to achieve good performance such as "feedback alignment" (Lilicarp et al, 2016) and "information alignment" (Kunin et al 2020). It is generally possible to encode multiple sequences in these models, and temporal scaling may be achieved easily simply by training on a diverse repertoire of sequences. Also, recent work suggests that with a reservoir of relevant dynamics it may be enough to train output weights only to achieve flexibility (https://arxiv.org/abs/2105.14108). These frameworks are quite flexible in the tasks that can be trained/executed, while the model the authors propose appears to be limited to executing particular action sequences; it is unclear how other kinds of tasks can be achieved in this framework. The authors should therefore discuss the tradeoffs in "baking in" certain prios via the proposed architecture.

Indeed, biologically plausible learning rules for neural networks date back to the 80s and 90s (e.g. the recirculation learning rule; Hinton & McClelland, 1988, Neural information processing

systems; O'Reilly, 1996, Neural Computation). More recently, within the realm of RNNs, local learning rules associated to random feedback (extending the fine-tuned feedback alignment in the work of Lilicrap) have shown to be able to learn complex sequential tasks (e.g., Murray, 2019, eLife). Furthermore, learning based on forwarding in time eligibility traces have displayed similar results (e.g. Bellec et al., 2020, Nat. Commun.). They are indeed quite flexible and can learn a variety of tasks (as demonstrated by Miconi, 2017, eLife). But while we agree with the Reviewer that such learning rules embedded in generic networks are powerful, and that temporal scaling may be achievable by training on a "diverse repertoire of sequences", such training schemes require immense amounts of data for the networks to display such rescaling, essentially requiring the network to have already seen the specific rescaled sequence that is now desired. The inductive bias induced in our architecture allows the network to separate timing from action identity and both from latent sequence order, allowing the network to immediately generate a desired sequence at a new tempo without any further training.

We now acknowledge several abilities of state-space models in the introduction, and clarify what are their limitations; we write on line 118:

"Recent work has shown that biological learning rules using local information can effectively learn complex (sequential) tasks[42–44] (albeit not as effectively as non-biological rules). State-space models can also implement a rudimentary form of temporal rescaling, in that they can rescale the timing of the execution of a single motor response[45], and iso-synchronous action sequences (e.g., index tapping at a steady rhythm)[34]. However, these models do not support temporal rescaling in the more general case (i.e., asynchronous action sequences). Furthermore, given their focus on cortical networks, these models do not address the growing evidence that action sequences unfold over multiple levels within cortico-basal ganglia-thalamic loops, with attractor state switches occurring in the prefrontal cortex[46] and action timing represented in the basal ganglia[2,4]."

The Reviewer also mentions the interesting work of Márton, Lajoie & Rajan (2020, arxiv), which touches on a distinct concept of flexibility. These authors focus on being able to use prior information gained by learning specific tasks, and how this information can be (partly) recycled to quickly learn a novel task. While the ability to extract such task primitives is undoubtedly powerful (and indeed this is a focus of some of our lab's other work), here we are focused on the ability to flexibly alter behavioral and neural dynamics without *any* further training, drawing on the concept of flexibility described in the work of Merhdad Jazayeri (Remington et al., 2018, Trends in Cognitive Sciences). As responded to a previous comment, our model was developed to account for flexibility in the production of precisely timed action sequences (e.g., Egger & Jazayery, Nature communications; Kornysheva et al., 2019, Neuron). Because of that, and following the no-free lunch theorem (Wolpert & Macready, 1997, IEEE Transactions on Evolutionary Computation) our model cannot account for other tasks usually solved through reservoir computing (Yang et al., 2019, Nature Neuroscience), in the same way that reservoir computing cannot account for what our model can. We now discuss the tradeoffs of our model in the limitation subsection of the discussion; we write on line 663:

"Moreover, our model (as others [22,28,67]) was specifically engineered to account for spatiotemporal sequences and how these may be flexibly manipulated. This in contrast to other instantiations[35,161] of RNNs (i.e. reservoir computing) that find natural solutions to diverse tasks involving distinct psychological processes (e.g., memory, time estimation, decision-making)."

If the novelty rather consists in achieving a wide-ranging amount of flexibility through a single model, then the authors should spell this out clearly and discuss tradeoffs/how they integrated different aspects.

As replied in previous comments, we now spell out more clearly the novelty in our work both on the theoretical level and what the model can achieve above and beyond other models.

The authors may also benefit from comparing to the following works: https://www.sciencedirect.com/science/article/pii/S0893608020303312?dgcid=rss_sd_all and https://www.nature.com/articles/s41593-019-0415-2

We thank the Reviewer for these references. We now incorporate the work of Márton, Schultz & Averbeck (2020, Neural Networks) in the discussion on line 647:

"Interestingly, Márton et al.[154] recently developed a RNN model of cortico-striatal interactions optimized to learn oculomotor sequences. Similar sequences were performed by awake monkeys while activity was recorded in their dorsolateral prefrontal and striatal areas. Learning to implement the correct actions for each sequence pulled apart the representational structure of action sequences in activity space both in the model and neuronal recordings. Whereas ordinal representations in our network were hardwired as orthogonal vectors in the RNN (in order to avoid interference), the work of Márton et al. suggests this may emerge naturally through learning."

The work of Nicola & Clopath (2019, Nature Neuroscience) proposes a spiking network that has the ability to compress theta sequences in sharp wave-ripples, and interestingly can reverse the order of the spatio-temporal sequence, which on its own is another instance of flexibility. We now add this work when referencing models that can perform temporal rescaling.

It is unclear how exactly the model is trained, where outputs are read out from and how, and which aspects of the model yield the purported flexibility. The authors argue at one point, for instance, that it is the "modularity" that "affords independent (and flexible) manipulation of sequence order and action timing" (p.5 bottom). But how is this conclusion supported by the reported results/figures? It is in fact unclear from the figures and explanations how the model is trained, how it executes actions (how actions are read out), and how/based on which aspects the flexibility emerges (more specific points below).

The training description of the model is located in the methods section (in particular within the subsection entitled "Learning in the ACDC model: Hebbian learning for order and Delta rule for time"). In order not to break the flow of the paper, we refer the reader to the methods for

training details (and also the full description of the model). We do however agree with the Reviewer that the specifics of our model with respect to learning and flexibility should be spelled out more clearly when we first describe our model in the result section; we now do so in our revised manuscript, line 160:

"[…]. This input targets a subset of RNN excitatory units, which cluster together via Hebbian learning, encoding the first latent state in the sequence (but not its specific action). In turn, the G (for Go) units in the BG learn (also via Hebbian learning) to link this RNN cluster to the appropriate action (blue arrow 1 in Fig. 1), allowing it to accumulate evidence for the first action in the sequence. The G node, part of a G-A-N triplet, projects excitatory connections to its correspondent A (for Action) node (blue arrow 2 in Fig. 1) which learns (via a delta rule) weight values for these projections to fine-tune the appropriate timing for this particular action. The A node represents motor thalamus, and its activation has two important consequences. First, it sends a thalamostriatal back-projection to excite the N node (blue arrow 3C in Fig. 1), which finally inhibits the G node via lateral connections from D2 to D1 medium spiny neurons[49]. Second, the thalamic A node triggers a transition in the RNN, via a combination of excitatory projections to another RNN cluster (blue arrow 3A in Fig. 1), and to a shared inhibitory neuron (blue arrow 3B in Fig. 1), consistent with evidence that thalamic units target both cortical excitatory and inhibitory neurons[50–52]. Thus, whenever an action is executed, the ratio of excitatory to inhibitory inputs to the RNN is perturbed in a way that induces a transition from the current cluster to the next cluster in line (targeted by the feedback projections of the current A node, blue arrow 3A in Fig. 1) to be expressed (see Methods for more details).

Learning takes place over fast and slow time scales. Hebbian learning is fast and unfolds within the dynamics of a trial (i.e., during the evolution of an action sequence). In contrast, the delta rule is slow and is implemented between trials, via a signed error computed through the discrepancy between the action timing provided by the tutor and the generated action. Action sequences are learned sequentially: the model learns to produce the first action at the appropriate time, then the second, and so forth. Sequential learning improves motor execution[53–57], and is at the base of several theoretical models of motor sequence learning[58–60].

At a higher-level, order is encoded as a sequence of attractor states represented by persistent activation in distinct excitatory RNN unit clusters (cell assemblies). These clusters do not represent the actions themselves but rather their abstract order; the specific actions to be executed are learned via RNN projections to the BG and their timing is encoded in the weights of topographic projections to the motor thalamus. To optimize precise action timing, the weights between action identity (G unit activity) and execution (timing for a given action conditional on G unit activity) are learned via supervised learning (i.e., delta rule), perhaps summarizing the role of cerebellum in error corrective learning. This allows us to model tasks in which a tutor provides feedback (e.g., [61]; see methods). Finally, feedback to the RNN from thalamic activity ultimately creates a cortico-basal ganglia loop. Each loop subtends the appropriate action order, identity and timing execution, allowing precisely timed action sequences to unfold. As we show below, our model architecture, allowing to uniquely encode

timing information in a distinct subset of the network (BG) than the one encoding order (PMC), will prove to display advantageous properties. In particular, being able to flexibly control, via external stimulation, the dynamics of the BG will result in a model displaying several temporal flexibility properties.

In many cases it seems like there is quite a wide gap between explanations/interpretations and what is actually depicted in the figures (specifics below). The manuscript would greatly benefit from updated Figure legends and explanations that carefully describe what is shown in the figures and how they support particular claims.

We have updated figure legends and have done our best (following the specific points made below) to close the gap between explanations and the depiction in our figures (see below for specific responses).

Specifics:

p.2: "We argue that this limitation emerges from the fact that order information and timing are typically stored in the same neural network weights" - how is this supported by your findings/what are the tradeoffs?

The general statement that order and timing is stored in the same weights in existing networks is simply an articulation of the fact that without an inductive bias in the architecture, learning induces a set of weights that minimize error for a particular sequence being trained, but do not enforce a factorization that would allow flexible rescaling in various ways we explore here. Our main argument is that the circuit evolved to perform such factorization, allowing action sequence features (i.e. action identity, sequence order, and action timing) to be manipulated independently. This instantiation is unique to our model, allowing us to independently modulate action timing with tempering with sequence order. Our results show exactly that: our model can learn any (iso-synchronous or asynchronous) arbitrary action sequence, shift it in time, rescale, and transform the timing of the actions in a flexible (i.e. not involving any further learning) manner. Therefore, because we can account for all these properties simultaneously and that separating order and time is unique to our model, we wrote the sentence highlighted by the Reviewer. We opted to maintain this sentence in the abstract, but we now clarify our logic throughout the text and show how our assertions are supported by our findings. Furthermore, we specify in the introduction (following our own previous work, see Franklin & Frank, 2018, eLife) that joint coding of task features only facilitates rigid forms of generalization and transfer. The same logic is applied here, if the order and the timing of a sequence are encoded by the same weights, manipulating one task feature will influence the other. Yet, musicians are able to independently manipulate aspects of action sequences (i.e. order and timing).

p.5: Comparison to state-space models - as explained in (1) this paragraph would benefit from a more careful evaluation and discussion of tradeoffs.

As suggested (by both Reviewers) we extensively changed the comparison to state-space models (see response to previous comment and line 118 in the revised manuscript).

p.5: "However, the mechanism for such compositionality in neural networks remains unknown" - This seems to suggest the main reason/novelty in this work consists in demonstrating how 'compositionality' is achievable. If so, then the manuscript should be restructured around this point and tradeoffs should be clearly explained (as mentioned in (1) - how many different types of tasks can be composed in this framework?). However, it seems that the type of flexibility achieved goes beyond just compositionality.

Yes, we agree with the Reviewer. We believe that the flexibility of our network goes beyond compositionality, and one of its strengths is that it can account for different types of flexible behavior simultaneously, as well as proposing how this is achieved via a biologically-inspired cortico-BG-thalamic loop model. As noted previously, we do agree that our model has been engineered (with biological constraints in mind) to mainly account for action sequence behavior (such as those involve in playing music, for instance; see response to previous comment). We therefore now acknowledge this limitation on line 663, and we delineate more clearly the novelty in our work in the introduction (see reply to previous comment)

p.5: "The modularity thereby affords independent (and flexible) manipulation of sequence order and action timing" – how is this supported by findings? What about previous work with modular networks?

A modular network allows us to encode time and order in distinct parts of the network, and hence manipulate the two pieces of information independently. As replied to the first specific comment, this is one novelty of our network, and it allows to manipulate timing information without disturbing order information. We show this in a series of simulation involving sequence flexibility. We now clarify this throughout the text (see novel additions to the text highlighted in green). To the best of our knowledge, our work is unique in providing a modular network that factorizes order and time and attributes these to distinct elements of a brain circuit.

p.6 Fig1: There is no information/explanation on biological plausibility of this architecture/training regime etc.

All the information can be found in the methods section. We deliberately situated this information in the methods in order not to disrupt the flow of the paper too much. As replied in a previous comment, we now have compacted this information in the beginning of the results section (when we first describe the model; see line 156). However, we refer the reader to the methods section on line 674 for a full description of the model.

p. 7, l.183-185/Fig2: How are actions trained? What are the inputs? Which part of the network

are actions read out from? More generally, how does learning proceed throughout the entire model - are the various component trained jointly or sequentially? What are the inputs/outputs of the various components?

See reply to previous comment.

p.8 Fig2: It is unclear how weights in D and E support the claim that there are "clusters" in the network. In F, it is unclear what the inputs are to the G and N nodes and how these are trained.

We agree that Fig. 2D does not necessarily support the result that clusters emerge from the network. The reason is that inputs to the RNN are random (but orthogonal; see methods). However, when inputs are sent to a random subset of RNN units, these units will cluster with one another according to equation 1 (see methods). If clusters did not form, we would not observe the persistent activation only within a subset of neurons (as displayed Fig. 7A), i.e. activation is maintained as an attractor state because these neurons are clustered and excite one another. We prefer to maintain randomness in the input to demonstrate that some form of structure in the input is not contingent to our results. Furthermore, we displayed Fig. 2D to show that some form of structure emerges in the RNN as learning progresses (given that initially no structure exists in the RNN; see methods). Information regarding the network is now added to the main text (see previous comments).

p.9 l.220: "We show that a previously learnt action sequence with temporal asynchrony can be flexibly reproduced" - based on Fig2A it seems that asynchrony is explicitly learned, so it would not be surprising that this will work (in the same way that supervised networks can achieve flexibility via training set). More detail on how exactly training proceeded/what actions were fed in as inputs would really help here.

We agree with the Reviewer, reproduction of a learnt sequence is a trivial result but still necessary to show as proof of concept and starting point for the following results. We have now embedded more information regarding details of the model in the main text (see line 160).

p.11 Fig3: Generally, it would be good to understand where these action sequences are read out from, what the inputs are, and what the other parts of the network are doing when throughout these sequences. It is unclear how D suggests 'Compression' as some of the actions seems to be aligned with the ones in B, while others seem slightly temporally shifted. Similarly, it is unclear how the right panel shows 'Dilation' as some of the actions appear in the exact same position as in the right panel of B and others only seem slightly shifted. Re F, it is unclear how this supports 'Compositionality' since the order is preserved and only the duration of particular actions seems extended.

One thing to note is that panel B and D are not perfectly aligned. But more importantly it is expected that some actions of the shifting simulations fall (in terms of timing) in the vicinity of the same actions for the rescaling simulations; this is a mere coincidence because of the

parameter values (i.e. the additional input time and the multiplicative input) we have chosen for these simulations. Another way of understanding this is that given a compression value, the original sequence can be shifted such that two contiguous actions (i.e. executed very close in time, as the green and cyan action) match those of the compression/dilation simulation. Note however, that the distance between the green and cyan peak is shorter/longer than in the shifting simulation compared with the compression/dilation simulation. Furthermore, the relative distance between action peaks is maintained as shown in Fig. 3E. Finally, there also is a very small shift in the rescaling simulation. This is because we give the multiplicative input to the entire set of G nodes; if we had avoided the input to the first G node, this very small shift would be avoided. However, we believe this way of implementing it is more parsimonious, and treat this small shift as a prediction of the model. We now have clarified this information in the results (line 305).

p.12 l 292/Fig 4: "via a learning mechanism" - what was the learning mechanism/how were these trained? How was multiplicative signal fed in, how were weights manipulated?

This simulation focused on the mechanistic properties of the network. The multiplicative signal selectively gains the G node of action two when it needs to be performed, thereby resulting in sustained activation. A similar result can arise from decreasing the weight from the action and NoGo node. We do not propose a learning mechanism for this but rather describe a mechanical property of the model. We now clarify these points in the results section (line 349):

"Note that we focus here on the mechanistic properties of the model, rather than proposing how the Action-NoGo weights may be learned."

p.13/Fig5: What was done to achieve the transformation from A to C? As in what combination of inputs/manipulations/training was necessary to achieve this?

Following learning described in the methods, we taught the network to produce the Thunderstruck song (Fig. 5A). We then used the tempo described in Fig. 5B as a multiplicative input to all G nodes. Applying this gain to the G nodes leads to the novel rhythm produced in Fig. 5C. We assume that the system has access to this tempo signal (i.e., the bossa nova rhythm that can be used by the system; note that this knowledge could easily be learned by a system that has experienced that tempo previously, indeed one could extract an abstract tempo by simply learning timings from the inhibitory neuron pool in the RNN). Hence, once the thunderstruck original rhythm has been learned, no additional learning is needed within the network to reproduce another rhythm; this is flexibly done via the multiplicative input (see line 312).

p.15/Fig7: How does part A show clusters in the network? How are switches "triggered" based on what we see in A? How are panels A and B related - are activations from the RNN fed into the G units? If so how are they wired, which part initiates an action? Perhaps this figure should be integrated with Fig2 for clarity.

Figure 7 shows that our model possesses similar dynamics as observed empirically, that is attractor state switches as the sequence unfolds in prefrontal cortex (e.g., Recanatesi et al., 2021, Neuron) and sequential sparse activation in BG (e.g., Gouvea et al., 2015). As responded in a previous comment, sustained activation in a subset of the RNN units arises from the fact that these neurons are clustered together. Input to the network is produce a single pulse, and sustained activation is an emergent property of the excitatory cluster (see methods section). The methods section further clarifies that indeed the RNN clusters feed to their corresponding G units, and the network learns to do so via hebbian learning (so the connections are not hard-wired). Given that this part mainly focuses on neurophysiological simulations, we prefer to keep it separate and not integrated with Fig. 2; as this allows us to provide some structure to the paper.

p.16, l.386: Based on the video and how activity evolved in PC space, it is not clear that these are in fact attractors - a fixed point analysis should accompany these analyses to support this conclusion. What initiates the switch from one state into another, assuming it is input driven? Also, the video shows 6 actions but only 5 distinct states in PC space - it seems some actions are mapped on top of each other, while others are not.

We define attractor states as sustained activation in a subset of RNN units that form a cluster and which do not get perturbed until there is another triggering event from A units to the RNN. We suspect the Reviewer is suggesting that these may not be attractors because they shift at each point in the sequence; but we meant that they are attractors in absence of further changes in the E-I balance of the RNN. To initiate a learned sequence, one input is sent to the first cluster as a single pulse. Given that each unit in the cluster projects excitatory connections to all the other units in the same cluster, sustained activation is achieved in that cluster; thereby, defining an attractor state as sustained activation without the need of a sustained input to the network (see Methods). Furthermore, switches from one attractor to another emerge from A nodes activation which projects back both to the RNN and to the shared inhibitory neuron. Indeed, A nodes activation perturbs the ratio of excitatory to inhibitory RNN input in a way that allows the current cluster to shut down and the following cluster to be expressed (see Methods). As for all previous comments we clarified this point when we first describe the network in the results section.
